# HEART RATE VARIABILITY ACTIVITY IN SOCCER ATHLETES AFTER A MUSCULOSKELETAL INJURY

**DOI:** 10.2340/jrm.v56.24969

**Published:** 2024-09-10

**Authors:** Gonçalo FLORES, Diogo MONTEIRO, Fernanda SILVA, Pedro DUARTE-MENDES

**Affiliations:** 1 ESECS – Polytechnic of Leiria, Leiria; 2 Research Centre in Sport, Health and Human Development (CIDESD), Vila Real; 3 Research Unit for Sport and Physical Activity, University of Coimbra, Coimbra; 4 Department of Sports and Well-being, Polytechnic Institute of Castelo Branco, Castelo Branco; 5 Sport Physical Activity and Health Research & Innovation Center, SPRINT, Santarém, Portugal

**Keywords:** autonomic nervous system, musculoskeletal disease, soccer athlete, sympathetic nervous system

## Abstract

**Objectives:**

The aim of this study is to analyse the adaptations of the autonomic nervous system after a musculoskeletal injury, obtained by measuring heart rate variability in athletes. It was hypothesized that there is an alteration in heart rate variability after a musculoskeletal injury.

**Study design:**

Cohort study.

**Subjects:**

15 semi-professional soccer players from three football teams, aged between 21 and 33 (mean age: 29.4 ± 3.31 years), with a recent musculoskeletal injury.

**Methods:**

Heart rate variability was collected using the Polar m200 and the chest strap H10 in two moments: within 72 h after the injury and between 5 and 7 days after full return-to-play.

**Results:**

Results show differences between T1 and T2 (*p* ≤ 0.05) in low-frequency power (n.u.) (*p* = 0.001) and high-frequency power (n.u.) (*p* = 0.001), in low-frequency/high-frequency ratio (*p* = 0.001) and in high-frequency power (ms^2^) (*p* = 0.017) measures. No statistical differences were found in low-frequency power (ms^2^) (*p* = 0.233). The low frequency power (n.u.) was significantly lower after injury compared with LF power (n.u.) values after full return-to-play. In high-frequency power there was a significant difference between both moments with high values after injury.

**Conclusions:**

The use of heart rate variability therefore seems to be promising to detect an imbalance in the autonomic nervous system and help clinical departments to identify a possible non-traumatic musculoskeletal injury. Further research should be performed considering a wide range of musculo-skeletal injuries and to establish baseline values of the athletes.

## LAY ABSTRACT

The aim of this study is to analyse the adaptations of the autonomic nervous system after a musculoskeletal injury, obtained by measuring heart rate variability in athletes. We hypothesized that there is an alteration in heart rate variability after a musculoskeletal injury. Fifteen male soccer players, aged between 21 and 33, with a recent musculoskeletal injury were recruited for the study. Heart rate variability was collected using the Polar m200 and the chest strap H10 in two moments. The use of heart rate variability seems to be promising to detect an imbalance in the autonomic nervous system, allowing an early identification of a possible non-traumatic musculoskeletal injury. Further research should be performed considering a wide range of musculoskeletal injuries.

Soccer is one of the most practiced sports around the world, with an increasing trend in the number of amateur and semi-professional athletes in recent years (1). This is a high-intensity sport that involves repeated movements such as changes of direction, sprints, jumps, and accelerations/decelerations, requiring physical and technical robustness capable of responding to these demands (2). Hence, with the increased number of athletes, the number of injuries has increased proportionately, not only in the professional leagues, but also at the semi-pro and amateur levels (1, 3).

The ethology of sports injuries is complex and multicausal, and arises from factors such as trauma, inadequate blood supply, muscle fatigue, excessive loads, or repeated movements to which athletes are exposed during sports practice (4, 5). In soccer, about 92% of injuries occur in the four big muscles of the lower limbs (hamstrings, quadriceps, adductors, and calves), leading to higher abandonment compared with many other sports (3).

After a musculoskeletal injury, the inflammatory process is one of the primary responses and the healing process is initiated through the autonomic nervous system (ANS) (4). The action from ANS influences the blood supply, transport of metabolic substances, and release of neuromediators involved in mechanotransduction in response to injury (4). Also, the increase in baroreflex activity and the released of inflammatory mediators could be responsible for activating nociceptors at the site of the injury (4, 6). Hence, an imbalance between the sympathetic nervous system (SNS) and the parasympathetic nervous system (PNS) may indicate a state of injury, with an increase in the sympathetic response and a decrease in parasympathetic activity, leading to changes in heart rate variability (HRV) indices (4, 7).

HRV is measured by the intervals between consecutive heartbeats. The evaluation of HRV is made through the QRS complex and the R–R intervals (interval between two consecutive R waves), more precisely through the electrical impulse that crosses the ventricles and includes the Q, R, and S waves, which reflect the activity of the sinoatrial node and the autonomic influence (8).

In the frequency domain, HRV is decomposed through oscillatory components of the heart, which allows the formation of a spectrum that divides oscillations into three main groups: very low frequencies (VLF; 0.0033–0.04 Hz), low frequencies (LF; 0.04–0.15 Hz), and high frequencies (HF; 0.15–0.4 Hz). VLF are influenced by physical activity, being a marker of sympathetic activity, LF represents sympathetic and parasympathetic activity, with a predominance of sympathetic activity, and HF reflect vagal tone (8–10). There is also the low-frequency/high-frequency ratio (LF/HF ratio) that reflects the general sympathetic-vagal tone, where an increase in the LF/HF ratio represents an increase in sympathetic activity and a decrease in parasympathetic activity, while a decrease in the ratio reflects an increase in parasympathetic activity and a decrease in sympathetic activity (7, 11).

The measurement of HRV can be performed using 24-h Holter electrocardiography. Yet, these devices present some limitations in terms of access costs, comfort, and freedom of movement (12, 13). Due to this, several devices were developed to assess HRV in a faster and more practical way, such as wireless heart rate monitors, which allow the calculation of R–R intervals with a resolution of 1 millisecond (ms) (12).

One of these devices is the heart rate monitor chest strap Polar H10 (Polar Electro Oy, Kempele, Finland), which allows the collection of HRV through a moistened elastic electrode strap placed under the chest muscle that identifies the electrical signals continuously transmitted by the heart and stores the data in a receiver (e.g., the Polar m200) connected via Bluetooth (14). This equipment has the same reliability and validity as the widely used Holter monitors in the assessment of HRV in the time domain, frequency domain, and in non-linear analysis, even being recommended when the assessment involves bodily movements (14).

## MATERIAL AND METHODS

With the increased number of injuries and the huge associated financial costs, clinical departments of the clubs are under additional pressure to keep athletes at their maximum level and, in case of injury, to recover them in the shortest possible time (15). Thus, it is essential for clinical departments to establish certain methods of early identification of a non-traumatic musculoskeletal injury, more precisely overuse injuries, and to manage the athlete at an early stage of injury, reducing their stop time (15). Therefore, the aim of this study is to analyse the adaptations of the ANS after a musculoskeletal injury, obtained by measuring HRV in athletes in the acute phase and after the return-to-play (RTP). We hypothesized that there is an alteration in HRV after a musculoskeletal injury, more specifically a decrease in HF power and LF power after injury and an increase in LF/HF ratio that tends to decline.

### Participants

A longitudinal study was carried out on a sample consisting of male individuals aged between 21 and 33 years (mean age: 29.4 ± 3.31 years). Only the participants who suffered a recent musculoskeletal injury were considered for the study. The sample was collected at three semi-professional soccer clubs in the central region of Portugal. Inclusion criteria were athletes diagnosed with a recent musculoskeletal condition (± 72 h post-injury) and without associated cardiac/respiratory (or other) health complications. Subjects were informed of the purpose of the study and signed an informed consent form. This study was conducted according to the Helsinki Declaration (16), and all procedures were approved. Fig. S1 shows the study flow diagram.

### Instruments

Heart rate variability was evaluated through Polar m200 and a polar heart rate monitor chest strap H10. The polar H10 was used to collect HRV measurements by detecting the R–R intervals, and the m200 sports watch was used as Polar H10 data logger. After data collection, all evaluations were exported to Polar Flow through the option “HRV data (CSV)” and analysed in the Polar Precision Performance Program software and Kubios HRV (Biosignal Analysis and Medical Imaging Group, Kuopio, Finland) in the frequency domain.

### Procedures

In a first phase, the clinical departments were contacted to ascertain availability for data collection. After acceptance, all participants filled out an informed consent form to guarantee privacy, confidentiality, and transparency of all collected data. Then, personal data (name, date of birth, contact information), the athlete’s medical history and the characteristics of the current injury were gathered to ensure the meet eligibility criteria. HRV was evaluated using the Polar m200 and the Polar H10. The present protocol was developed based on previous studies (17–20).

Data collection was performed in two moments. The first moment was within 72 h after the injury (T1) and the second moment was between 5 and 7 days after the full RTP (T2). Recordings were preferably carried out at the same time. The Polar m200 was placed on the subject’s left wrist and the Polar H10 around the chest, and athletes remained in a standing position with their arms at their sides. Before starting the assessment, the participant remained at rest for 5 min in a chair. The HRV assessment through Polar m200 and Polar H10 lasted 8 min and was always performed on the subject’s left arm when standing. For study purposes, only the values obtained between the 2nd and 8^th^ min were considered for analysis. Fig. S2 presents a schematic of the study design.

The Polar Precision Performance Program Software and Kubios (Biosignal Analysis and Medical Imaging Group, Kuopio, Finland) were used to import, analyse, and store the data (20–22). The analysis of HRV was performed in the frequency domain, dividing the oscillations into three groups: very low frequency (0.0033–0.04 Hz), low frequency (0.04–0.15 Hz) and high frequency (0.15–0.40 Hz), and expressed in absolute values (ms^2^) and normalized units (n.u.) (12, 23). All collected and analysed data follow the recommendations of the Task Force of the European Society of Cardiology and North American Society of Pacing and Electrophysiology (24).

### Statistical analysis

*Preliminary analysis.* Initial data analysis excluded one athlete because he was statistically determined to be an outlier. A priori power analysis through G^*^Power (3.1.9.2) (25) was used to determine the required sample size considering the following input parameters: effect size d = 0.8; α = 0.05; statistical power = 0.9. The required sample size was 15, which was respected in the present study. The suggested size effect and remaining parameters were defined according to similar studies that evaluated changes in HRV during exercise protocols (26).

*Statistical analysis.* Descriptive statistics – including mean and standard deviation – were calculated for all variables under analysis. A Shapiro–Wilk test (*n* < 50) was performed to analyse data distribution, and *p* > 0.05 was considered as a normal distribution.

To analyse the differences between the two phases (after injury and after RTP) in HF power (n.u.) and LF power (n.u.) a paired samples t-test was used due to normal distribution in the Shapiro–Wilk test, and to analyse the LF/HF ratio, HF power (ms^2^) and LF power (ms^2^) a Wilcoxon test was used due to non-normal distribution in the Shapiro–Wilk test. Data are presented as mean ± SD for variables with normal distribution and median (interquartile range) for non-normal distribution variables.

To compare the pre- and post-intervention, the magnitude of the effect was calculated using Cohen’s d effect size and was interpreted as follows: < 0.20 (small), 0.20–0.79 (moderate) and > 0.80 (large) (27). In the variables with non-normal distribution, the effect size was calculated based on the eta square value (η^2^) according to the formula η^2^ = Z^2^/N (28, 29). Data analyses were performed using IBM SPSS Statistics 24 (IBM Corp, Armonk, NY, USA). For all statistical procedures, the considered level of significance was 0.05.

## RESULTS

### Characteristics of subjects

Fifteen semi-professional athletes (29.4 ± 3.31 years old) successfully completed the study, presenting nine types of musculoskeletal injuries, of which six previously had a similar injury. Fourteen athletes performed three training sessions per week and one athlete performed four training sessions. Table I presents the baseline characteristics of all participants included in the present study.

**Table I:** Baseline characteristics of the study participants included in the analysis

Variables	All (*n* = 15)
Age, years, mean ± SD	29.4 ± 3.31
Regular medication, *n* (%)	0 (0)
Type of injury, *n* (%)	
Muscle tear isquiotibiales	6 (40)
Muscle tear rectus femoral	2 (13.3)
Pubalgia	1 (6.7)
Tibiotarsal sprain	1 (6.7)
Knee sprain	1 (6.7)
Calcaneal trauma	1 (6.7)
Acromioclavicular traumatic injury	1 (6.7)
Peroneal fracture	1 (6.7)
Glenohumeral dislocation	1 (6.7)
Previous similar injury, *n* (%)	
Yes, *n* (%)	6 (40)
No, *n* (%)	9 (60)
Training sessions weekly, *n* (%)	
Three, *n* (%)	14 (93.3)
Four, *n* (%)	1 (6.7)

SD: standard deviation.

Table II indicates the differences between T1 and T2 in the studied variables. Differences between T1 and T2 (*p* ≤ 0.05) in LF power (n.u.) and HF power (n.u.), in LF/HF ratio and in HF power (ms^2^) were found. No statistical differences were found in LF power (ms^2^).

**Table II:** Differences between T1 and T2 on LF power (n.u.), HF power (n.u.), LF/HF ratio, LF power (ms^2^), and HF power (ms^2^) outcomes calculated with *t*-test and Wilcoxon test

Outcome	*n*	T1 Mean ± SD	T2 Mean ± SD	*p*-value	η2	Effect size	95% CI Median (IQR)
LF power (n.u.)	15	69.48 ± 17.27	83.65 ± 10.37	0.001		0.995	–0.078 –2.068
HF power (n.u.)	15	30.49 ± 17.24	16.32 ± 10.35	0.001		–0.996	–2.069 –0.077
LF/HF ratio (%)	15	2.81 (3.38)	5.42 (10.37)	0.001	0.699	3.044	
LF power (ms^2^)	15	828.50 (650.51)	1,162.30 (991.29)	0.233	0.095	0.648	
HF power (ms^2^)	15	205.36 (911.16)	218.18 (380.29)	0.017	0.379	1.563	

SD: standard deviation; IQR: interquartile range; LF power: low-frequency power; HF power: high-frequency power; LF/HF ratio: low-frequency/high-frequency ratio.

## DISCUSSION

The objective of the present study was to analyse the activity of the sympathetic nervous system and the parasympathetic nervous system – two branches of autonomic nervous system – after a musculoskeletal injury and after full return-to-play in athletes, using heart rate variability. The use of HRV could be useful for clinical departments to identify a possible non-traumatic musculoskeletal injury in the early stages, reducing stop time, or to facilitate a safer RTP after injury.

Several studies have already demonstrated the role of the ANS in response to musculoskeletal injuries (4, 30–32). The afferent vagal neurons communicate the somatic tissue damage to the brain, which results in the activation of the efferent vagal fibres increasing blood supply, transport of metabolic substances, and the release of proinflammatory cytokines (4, 18, 30, 33). HRV is a rapid and non-invasive tool that can be used to detect an imbalance between the sympathetic and parasympathetic nervous system (18, 32), in which a reduction in HRV is associated with early signs of somatic tissue distress, even prior to pain or fully developed injury (19, 32, 34). These findings agree with Jewson et al. (31), who associated alterations in HRV with the development of a tendinopathy.

In the analysis of HF power, there was a significant difference between the two moments, with high values in HF power after injury when compared with the results obtained after full RTP. It is noted, however, that this finding does conflict with previous studies, which refer to a decrease in HF power after injury, due to an increase in SNS activity and a decrease in PNS activity (17, 22, 34). This reduction was also observed by Hellard et al. (18) during weeks characterized by an increased risk of muscular affections in elite swimmers and in response to overtraining stimulus. The somatic tissue damage leads to ANS stimulation and higher sympathetic activation that regulates the healing process, leading to a decrease in parasympathetic activity, and a decrease in HF power (4, 18, 32, 34). These divergences could be related to the different evaluation times of athletes and to the different types of injuries between studies, as all cited studies focus on concussion.

In the current study, LF power (n.u.) was significantly lower after injury compared with LF power (n.u.) values after full RTP and these results corroborated the studies of Gall et al. (35), Bishop et al. (17) and La Fountaine et al. (36). Despite that, Hellard et al. (18) and Senthinathan et al. (22) refer to an increase in LF power, due to higher activation of the sympathetic nervous system.

In the analysis of the LF/HF ratio, significant differences were found between the two moments, with low values of the LF/HF ratio after injury, which tends to increase after full recovery. This has not been consistently reported in prior literature. Some studies (21, 22, 34) demonstrated a higher LF/HF ratio after injury that tends to decline with time and recovery. This difference could be justified with the results found in HF power, as the ratio is dependent on the values of LF and HF power.

### Limitations

Although the present study contributes to our understanding of HRV behaviour after a musculoskeletal injury, it has some limitations. Among them is the use of different protocols to collect HRV in the literature. More specifically, the timing of assessment after injury, the number of assessments, and the form of evaluation (e.g., some studies collect data at rest, standing, or during submaximal exercise). Another limitation is that all studies in the literature focus on concussion and there are none that investigate HRV behaviour after other types of musculoskeletal injuries, such as muscle tears or pubalgia, which were presented by some subjects in this study.

Moreover, longitudinal and/or experimental studies are needed to further examine the effects of the analysed variables, to increase our knowledge regarding HRV, and to use them to detect early signs of somatic tissue distress, so reducing athlete stop time, or to help clinical departments to facilitate a safer RTP. The inclusion of a group control and baseline assessment (before the injury) would be useful to better understand this relationship. Furthermore, we cannot control some conditions, such as stress or pain. Therefore, we suggest future studies considering a wider range of musculoskeletal injuries and sports modalities.

### Clinical implications

The analysis of HRV in athletes could help clinical departments to detect the development of an overuse musculoskeletal injury and facilitate a safer return-to-play.

### Conclusion

The present study suggests that, after injury, an alteration in HRV is observed in athletes. Specifically, a reduction in LF power and LF/HF ratio, and an increase in HF power was observed after injury. Despite the inherent limitations, our findings have important clinical implications because this is one of the first studies to investigate HRV alterations, in the frequency domain, after different types of musculoskeletal injuries.

## Supplementary Material

HEART RATE VARIABILITY ACTIVITY IN SOCCER ATHLETES AFTER A MUSCULOSKELETAL INJURY
