# Supplementary material for: HEART RATE VARIABILITY ACTIVITY IN SOCCER ATHLETES AFTER A MUSCULOSKELETAL INJURY
Source: J Rehabil Med. 2024 Sep 10;56:24969. doi: 10.2340/jrm.v56.24969 (PMC11407110; doi:10.2340/jrm.v56.24969)
Supplement: HEART RATE VARIABILITY ACTIVITY IN SOCCER ATHLETES AFTER A MUSCULOSKELETAL INJURY [file JRM-56-24969-s1.pdf]

Supplementary material has been published as submitted. It has not been copyedited, or typeset by Journal of Rehabilitation Medicine

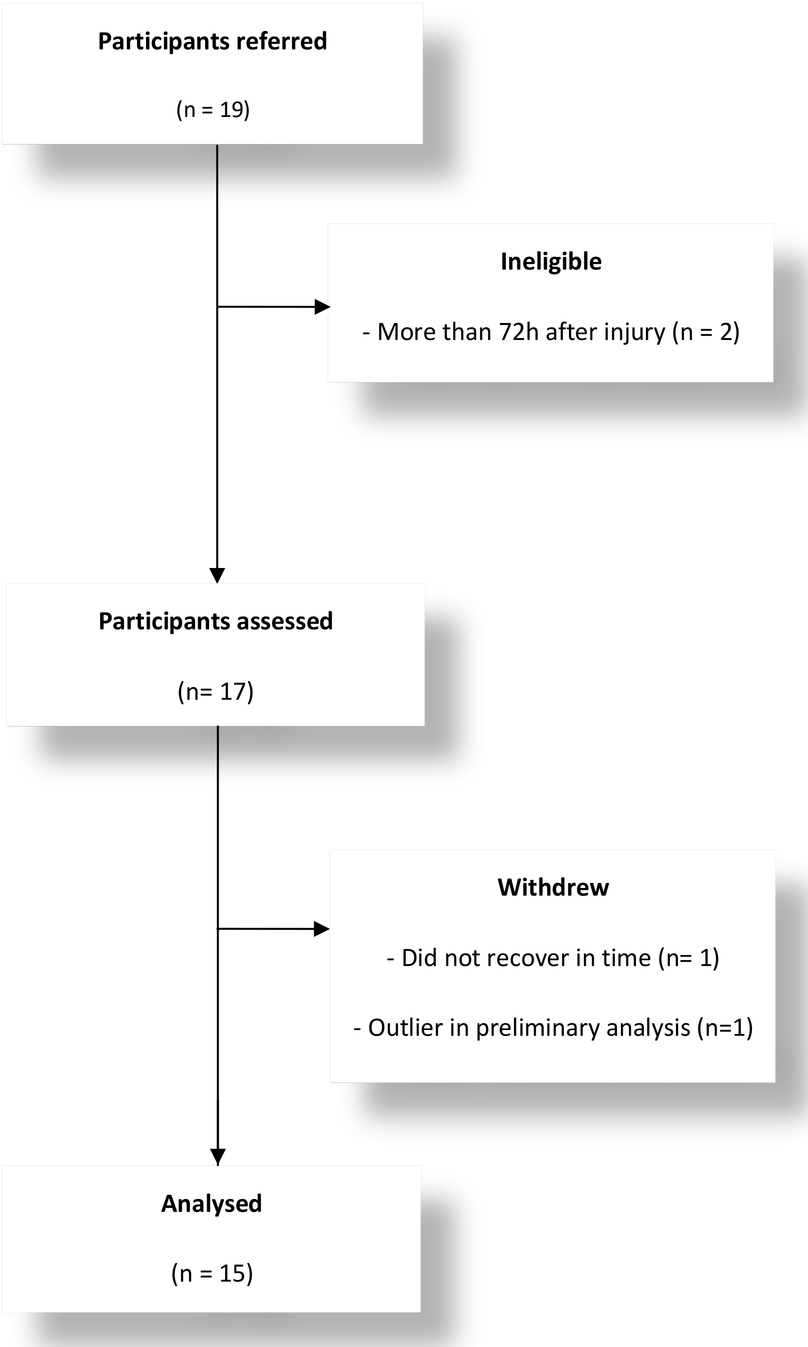

Figure S1. Study flow diagram.

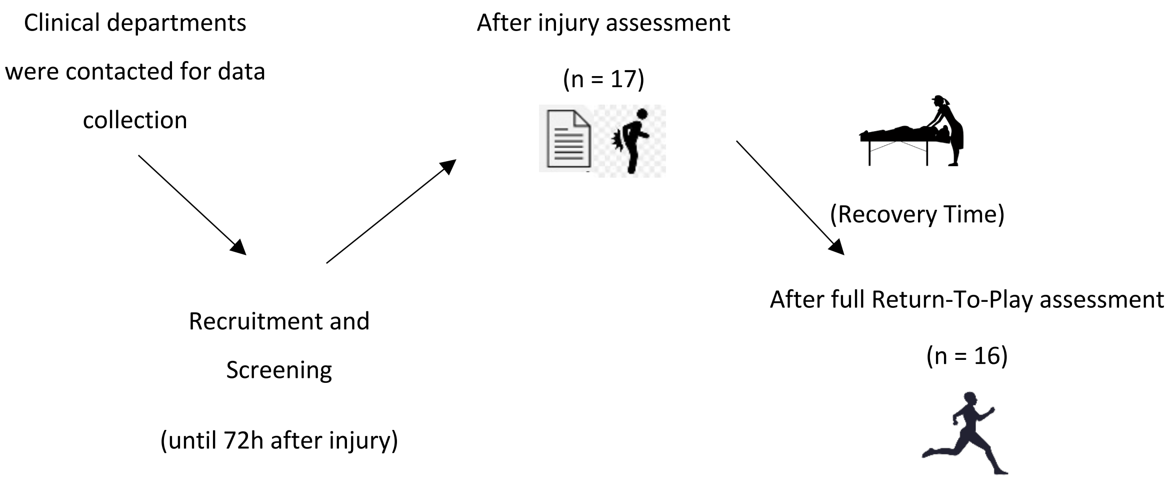

Figure S2. Schematic of study design.
